# Editorial: The emerging role of liquid biopsies in hematologic disorders

**DOI:** 10.3389/fonc.2024.1437621

**Published:** 2024-06-21

**Authors:** Ioannis Ntanasis-Stathopoulos, Daniela Drandi

**Affiliations:** ^1^ Department of Clinical Therapeutics, School of Medicine, National and Kapodistrian University of Athens, Athens, Greece; ^2^ Department of Molecular Biotechnology and Health Sciences, Hematology Division, University of Torino, Torino, Italy

**Keywords:** cell-free, liquid biopsy, lymphoma, myeloma, leukemia

In the field of hematologic diseases, the advent of liquid biopsies has witnessed a significant shift towards precision medicine. The potentiality of this minimally invasive procedure, that entails collecting a sample of bodily fluids, such as blood, urine, or cerebrospinal fluid, instead of performing a traditional tissue biopsy, has great promise in revolutionizing the way we diagnose, monitor, and manage hematologic disorders.

The current research topic gives a thorough analysis of liquid biopsy applications in investigating novel techniques and methods as well as highlighting the clinical utility of liquid biopsy, specifically exploring its significance as a diagnostic, prognostic, or minimal residual disease (MRD) monitoring biomarker.

In the conventional approach, the identification and surveillance of hematologic diseases have predominantly depended on the utilization of invasive tissue biopsies and imaging tests. Nevertheless, these methodologies frequently exhibit constraints such as their invasive nature, associated risks, and the incapacity to capture the dynamic nature of the disease. Hematologic malignancies including leukemia, lymphoma, and multiple myeloma, which diagnosis still requires histological confirmation by bone marrow (BM) aspirate, shows great potential for the utilization of liquid biopsies. These tumors exhibit genetic diversity and clonal evolution, which poses significant difficulties in properly monitoring and treatment. The source of biofluid fraction, such as extracellular vesicles (EVs) or the circulating-free compartment (cfDNA or cfRNA) presents in liquid biopsies, provides a comprehensive and dynamic perspective on tumor genetics, identify specific targets for therapeutic intervention enabling clinicians to effectively monitor the progression of disease, and assess the efficacy of treatments.

Concerning this, the Talotta et al. and Zerdan et al. reviews, gave a very comprehensive and exhaustive overview of methods and applications of liquid biopsies as diagnostic and prognostic markers in many types of lymphoma and other hematological malignancies. While, Haider et al. report a proof-of-concept study on using whole-genome sequencing for disease monitoring in B-cell lymphomas. The study presents a clinically feasible approach by multiplex digital PCR to quantify circulating tumor DNA (ctDNA) burden as a measure of MRD in diffuse large B-cell Lymphoma (DLBCL) and follicular lymphoma, showing a higher sensitivity, compared to the routine radiological imaging approach.

Moreover, not only ctDNA can recapitulate the mutational profile of tissue biopsies and identify mutations not detected in the tissue. For instance, the study from Decruyenaere et al. describes a unique cfRNA signatures of blood plasma, compared to tissue samples, in DLBCL, primary mediastinal B-cell lymphoma (PMBCL) patients and healthy donors. This study highlights the potential of plasma-derived gene expression profiling to accurately identify diagnostic, cell-of-origin, and prognostic cfRNA biomarkers for diagnosing and disease monitoring of lymphoma patients.

It is worth mentioning that the term “MRD” denotes the existence of residual cancer cells either during or after therapy, frequently evading detection through traditional means. Residual cells have the potential to function as a reservoir for future recurrence, whereas achieving MRD negativity has been associated with improved outcomes in patients with hematologic malignancies. The easily collection at multiple time points and the capacity of liquid biopsies to identify small amounts of circulating tumor DNA or cells, make them a viable method for early identification of MRD and proactive management. This approach has the potential to enhance patient outcomes and increase survival rates.

As reported by Yu et al., MRD research has seen a steady rise over the past two decades. Considering the rapid development and increasing applications, it is likely that MRD research will continue to advance, and liquid biopsy holds great promises for becoming more widely adopted in the diagnosis and MRD monitoring providing valuable information for personalized treatment strategies.

Lastly, liquid biopsies have significant potential in detecting and longitudinal monitoring of MRD both in lymphoid and myeloid malignancies. Indeed, Allam et al. provide an overview of the use of liquid biopsies and MRD assessment, for monitoring treatment response and predicting prognosis, also in myeloid malignancies, including acute myeloid leukemia and myelodysplastic syndromes.

Although the considerable potential, the extensive use of liquid biopsies in the clinical setting encounters many obstacles. The establishment of standardized protocols for sample collection, processing, and analysis is of utmost importance in order to guarantee the reproducibility and reliability of research findings. In addition, the analysis of liquid biopsy data necessitates the utilization of advanced bioinformatics tools and algorithms that possess the ability to differentiate genuine signals from undesirable background noise and abnormalities, such as the clonal hematopoiesis of indeterminate potential.

Furthermore, it is imperative to address concerns regarding cost-effectiveness and reimbursement in order to ensure the accessibility of this technology to all eligible patients. Despite the current limitations, the ongoing investigations will result in advancements in this field and support the precision medicine in the clinical practice.

Finally, liquid biopsies in hematologic diseases show great potential for the future as continuous research and technical progresses are expected to overcome limitations. The incorporation of additional omics data, such as proteomics and metabolomics, has the potential to significantly improve the diagnostic and prognostic effectiveness of liquid biopsies. Machine learning and artificial intelligence algorithms provide the capability to efficiently assess extensive quantities of intricate data, hence facilitating the development of individualized treatment methods that are customized to the distinct molecular profiles of each individual patient.

Moreover, as our comprehension of the tumor microenvironment and immune response progresses, liquid biopsies provide a means to observe these dynamic interactions, hence facilitating the advancement of immunotherapies and tailored treatments. The integration of liquid biopsy analysis with additional diagnostic modalities, such as imaging and conventional tissue biopsies, has the potential to offer a holistic and synergistic strategy for disease management. A key element is the incorporation of liquid biopsies in the design of clinical trials. Clinical endpoints and treatment decisions based on the results of liquid biopsies, such as cell-free DNA/RNA or circulating tumor cells, are crucial to be evaluated in randomized clinical trials in order to formulate the future standards of care.

## Author contributions

IN-S: Writing – original draft, Writing – review & editing. DD: Writing – original draft, Writing – review & editing.

